# Preliminary Studies on Validation of Calu-3 Cell Line as a Model for Screening Respiratory Mucosa Irritation and Toxicity

**DOI:** 10.3390/pharmaceutics6020268

**Published:** 2014-06-13

**Authors:** Chibueze Ihekwereme, Charles Esimone, Di Shao, Remigius U. Agu

**Affiliations:** 1 Department of Pharmacology and Toxicology, Nnamdi Azikiwe University, Awka 420281, Nigeria; E-Mail: cp.ihekwereme@unizik.edu.ng; 2 Department of Pharmaceutical Microbiology and Biotechnology, Nnamdi Azikiwe University, Awka 420281, Nigeria; E-Mail: co.esimone@unizik.edu.ng; 3 Biopharmaceutics and Drug Delivery Laboratory, College of Pharmacy, Dalhousie University, Halifax, NS B3H 3J5, Canada; E-Mail: dishao@hotmail.com

**Keywords:** Calu-3 cells, MTT, toxicity prediction, mucosal toxicity

## Abstract

There is need to develop reproducible methods and experimental models for screening mucosal irritation and toxicity for drugs and pharmaceutical excipients. The aim of this study was to validate Calu-3 cell line as a model for screening respiratory irritation and toxicity of drugs and excipients. Eighteen test compounds were selected according to their irritation potential and European Centre for the Validation of Alternative Methods (ECVAM) guidelines. Cell toxicity and irritation was determined using MTT assay. Data analysis and interpretation were done using modified ECVAM approach; where replicate values met acceptance criteria if percent relative standard deviation (RSD) of the raw data is <18%. Compounds with mean relative viability values of 50% and below were classified as irritant (I); those above 50% were non-irritant (NI). At low concentration (0.2% *w*/*v*) and 1 h incubation, the Calu-3 cell culture model accurately predicted the toxicity of most test compounds. The specificity of our proposed model (percentage of *in vivo* non-irritants correctly predicted), concordance (percentage of compounds correctly predicted) and sensitivity (percentage of *in vivo* irritants correctly predicted) at 0.2% *w*/*v* and 60 min exposure were 100%, 72%, and 44%, respectively. In conclusion, the Calu-3 cell line in conjunction with MTT assay appears to be a potentially useful tool for screening drugs and excipients for respiratory mucosa irritation and toxicity. However, as the data reported in this study were solely based on MTT assay, additional studies are needed using other toxicity-/irritation-indicating methods to confirm the observed trend.

## 1. Introduction

There are concerns about the use of animals in research experiments. Russell and Burch in 1959 introduced the 3R principles [1]. The 3Rs refer to ***R***eplacement, ***R***eduction and ***R***efinement of animal use in scientific experiments. The principle seeks to minimize the use of non-human vertebrates in research. On 11 March, 2013, the European Union discontinued the use of cosmetics or their ingredients tested on animals [2]. There is need to develop reproducible methods and experimental models for screening mucosal irritation and toxicity for drugs and pharmaceutical excipients that are subject to high throughput screening. Regulatory authorities require safety profile of chemicals, pharmaceuticals, and personal care products before they are available for public use [3]. There is no generally accepted *in vitro* cell culture model for assessing respiratory irritation [4]. The Draize rabbit eye test has been the standard test accepted by regulatory authorities for assessment and classification of the capacity of chemicals to damage the mucosa of the eye [5]. It is a whole animal test that involves direct application of test substance to the conjunctiva sac of one of rabbit’s eyes, whereas the untreated eye serves as control [6]. Similarities exist between the eye and the respiratory mucosa. Both are protective layers capable of producing mucous [7]. Both eye and respiratory mucosal layers generally produce mucus that contains mucin as a key component. Slug mucosal irritation (SMI) and bovine corneal opacity and permeability (BCOP) assays have been used to assess ocular and respiratory mucosa irritation [8,9,10,11].

The slug mucosal irritation (SMI) test method was developed as an alternative test for screening toxicity of mucosal surfaces using the invertebrate, *Arion lusitanicus* as a model organism [12]. Twenty eight substances selected from the eye irritation reference chemical data bank of the European Centre for Ecotoxicology and Toxicology of Chemicals (ECETOC) were used to screen the SMI model as there were no reference standards for screening mucosal toxicity of chemicals [12]. The rationale for the selection and application of this model to the respiratory cells is understandable considering the similarities between ocular and respiratory mucosa. However, the model appears to lack high throughput screening capability that is possible with cell culture models. It is a non-human model and may not physiologically reflect specific tissue response to toxins unique to different respiratory regions. Based on these reasons, we decided to validate the Calu-3 cell culture as a model for screening respiratory mucosa toxicity.

Calu-3 cells are well-characterized cell line derived from bronchial adenocarcinoma of the airway [13,14]. The cells are popular in respiratory cell research because they demonstrate properties of the bronchiolar epithelium and are unique in a number of ways. The Calu-3 cells have characteristics of both serous and mucus cells, can be cultured as a flat sheet, and respond to secretagogues that regulate the glands *in vivo*. They form polarized monolayers with tight junctions and express several acinar cell markers [15]. The cells have been used in studies involving drug transport, respiratory drug deposition, metabolism and bioactivation of toxins, and toxicity of inhalants, nanoparticles, and surfactants [14,16,17,18]. Based on these reports, the popularity, and accessibility of the cell line, we chose it for our studies.

## 2. Experimental Section

### 2.1. Chemicals

Isopropanol was supplied by Fischer Scientific (Ottawa, ON, Canada). Tissue culture materials including Dulbecco’s Modified Eagle’s Medium–Ham’s F-12 (DMEM/F-12), fetal bovine serum (FBS), phosphate buffered saline (PBS), penicillin/streptomycin, Glutamax^®^, and phosphate-buffered saline-trypsin were purchased from Invitrogen (Burlington, ON, Canada). Human bronchial/sub-bronchial gland cell line (Calu-3) was purchased from American Type Culture Collection (Manassas, VA, USA). Irritation reference chemicals, classes, and sources are listed in Table 1.

### 2.2. Cell Culture

The Calu-3 cells were used at passages 5–10. They were cultured in 96-well plates (Fisher Scientific) in 1:1 DMEM/F-12 supplemented with 10% FBS, 1% Glutamax^®^, 100 U/mL penicillin, and 100 mg/mL streptomycin using our previously described method [14]. The tissue culture medium was changed every two days. The cells were maintained at 95% O_2_ and 5% CO_2_ environment and were used for experiments at 70% confluency.

### 2.3. Chemical Exposure to Calu-3 Cells

Irritant selection, concentration and exposure time were based on mucociliary clearance time and published work in which the compounds were used. As the average respiratory mucociliary clearance time is about 20 min, we decided to investigate the effect of the compounds following 15, 30, and 60 min exposure. In order to provide a basis for comparison with other published work using other models three concentrations of the chemicals were selected (0.2%, 0.4% and 1.0%). To initiate the experiments, the cells were washed 3 times with PBS and allowed to equilibrate in 100 µL of buffered DMEM/F-12 (without phenol red) for 30 min. The medium was then removed and replaced with 100 µL of 0.2% or 1.0% solutions of test substances in DMEM/F-12 and were incubated for 15, 30, and 60 min, respectively in 5% CO_2_/95% O_2_ incubator maintained at 37 °C. Cells were observed under the microscope for detachment before discarding the test solutions. Subsequently, the effect of the test compounds on the cells was determined using MTT assay.

**Table 1:** Compounds used for toxicity studies.

S/N	Chemical Name	Chemical Class	European Union Irritation Class			CAS Number	Log *p* *
			Non-Irritating	R36 Irritating to Eye	R41 Risk of Serious Damage to Eye		Octanol–Water
1	PEG 400	Alcohol	√			25322-68-3	−1.21
2	3-Methoxy-1,2-propanediol	Alcohol	√			623-39-2	−1.20
3	Glycerol	Alcohol	√			56-81-5	−1.76
4	PEG 600	Alcohol	√			25322-68-3	−1.21
5	2-Methyl-1-pentanol	Alcohol	√			105-30-6	1.75
6	Anhydrous ethanol	Alcohol	√			64-17-5	−0.30
7	Cyclohexanol	Alcohol			√	108-93-0	1.23
8	Tween 20	Surfactant	√			9005-64-5	2.39
9	Sodium dodecyl sulphate USP	Surfactant		√		151-21-3	1.60
10	Triton X-100	Surfactant		√		9002-93-1	4.15
11	Cetylpyridinium bromide	Surfactant			√	140-72-7	1.83
12	Toluene	Heterocyclic	√			108-88-3	2.73
13	Imidazole	Heterocyclic			√	288-32-4	−0.08
14	Methyl isobutyl ketone	Ketone	√			108-10-1	1.31
15	Acetone	Ketone		√		67-64-1	−0.24
16	Sodium hydroxide	Inorganic chemical			√	1310-73-2	0
17	Sodium oxalate	Carboxylic acid salt			√	62-76-0	−0.26
18	4-Fluoroaniline	Amine			√	371-40-4	1.15

* The Log *p* values were from [19,20].

### 2.4. MTT Assay

Cell viability screening was based on cellular mitochondrial dehydrogenase (MDH) activity, measured by MTT reduction and conversion to blue formazan salt that was quantified after extraction from cells [21]. MTT was used for predicting respiratory irritation because several studies have shown correlation between reduction in cell viability, decrease in epithelial electrical resistance and increases in biomarkers such as IL-1α and IL-1β. Cells grown in 96-well plates were used for the studies. After exposure of the cells to the solution of test compounds, the solutions were discarded and 100 μL of MTT (5.0 mg/mL) added to each well and incubated for 3 h at 37 °C. Subsequently, the MTT solution was removed and replaced with 100 μL isopropyl alcohol. The plates were then wrapped in aluminium foil and incubated for 2 h at 37 °C in order to extract formazan from the cells. Extracted formazan salt was quantified spectrophotometrically at 570 nm using Synergy™ HT multimode microplate reader (BioTek Instruments Incorporated). The absorbance of isopropyl alcohol (100 μL) was used as blank. The reduction of cell viability in treated cells was compared to negative control (cells incubated with DMEM/F-12) and expressed as a percentage. Benzalkonium Chloride (5.0%) was used as positive control since it is known to be irritant/toxic to airway cells and has been used in similar studies [22,23,24].

### 2.5. Data Analysis

Data acceptance criteria, calculation steps, interpretation and prediction model was done using the ECVAM Skin irritation Validation Study as contained in Standard Operating Procedure of Validation of the EpiSkin™ Test Method ^15 min–42 h^ for the prediction of acute skin irritation of chemicals [25]. This was chosen because the model, as well as the cell viability assay method (MTT) has been accepted by intergovernmental agencies as being able to distinguish between irritants and non-irritants [25]. However, we modified drug exposure time to reflect respiratory route of administration (15–60 min). In our experiments, replicate values (*n* = 4) from each well, both for the treated and controls met the acceptance criteria if the percent relative standard deviation value (% RSD) of the raw data is <18%. The negative control (NC) met the acceptance criteria if the % RSD of the percent viability is <18%. The positive control (PC) met the acceptance criteria if the mean viability and standard deviation expressed as percentage of the NC, is <40% and <18%, respectively [26]. Test compounds with mean relative viability values of 50% and below were considered irritant (I); those above 50% were considered non-irritant (NI) according to the ECVAM protocol. The concordance (percentage of compounds correctly predicted), sensitivity (percentage of *in vivo* irritants correctly predicted) and specificity (percentage of *in vivo* non-irritants correctly predicted) were calculated [12].

## 3. Results and Discussion

### 3.1. Results

As there were no reference standards for screening respiratory mucosa irritation and toxicity, the reference chemicals used were selected from the ECETOC data bank for eye irritation. Twenty-eight compounds that cover entire irritancy range were selected [12], but eighteen (9 irritants and 9 non-irritants) were used mainly due to solubility problems. The target maximum solubility for our studies was 1.0 *w*/*v*% and compounds with lower solubility in Dulbecco’s Modified Eagle’s Medium–Ham’s F-12 (DMEM/F-12) were excluded. The test chemicals included seven alcohols (6 non-irritants and 1 irritant), four surfactants (1 non-irritant and 3 irritants), two heterocyclic compounds (1 non-irritant and 1 irritant), two ketones (1 non-irritant and 1 irritant), one inorganic compound (irritant), one carboxylic acid salt (irritant), and one amine (irritant) (Table 1).

The result obtained after 60 min exposure of the Calu-3 cells to 0.2% test solutions showed that all the alcohols had viabilities greater than 50% (Table 2). The values were PEG 400 (91.5 ± 4.6); 3-methoxy-1,2-propanediol (92.6 ± 4.1); glycerol (85.6 ± 8.7); PEG 600 (85.9 ± 13.4); 2-methyl-1-pentanol (95.3 ± 13.1); anhydrous ethanol (99.1 ± 3.9); and cyclohexanol (89.9 ± 14.3). Most of the tested surfactants were remarkably toxic to the Calu-3 cells. Other than Tween 20 (NI) that resulted in 57.1% ± 8.1% viability, 0.2% *w*/*v* of sodium dodecyl sulphate (R36), Triton X-100 (R36) and cetylpyridinium bromide (R41) killed more than 90% of the cells. The heterocyclic compounds: Toluene (NI) and imidazole (R41) had no significant effect on the cell viability. Similar results were observed for the ketones (methyl isobutyl ketone (NI) and acetone (R36)). Whereas the 0.2% *w*/*v* of the inorganic compound (sodium hydroxide, R41) killed about 95% of the cells, the tested carboxylic acid (sodium oxalate, R41) and amine (4-fluoroaniline, R41) resulted in 65.4 ± 4.0 and 114.9% ± 5.9% viability, respectively.

**Table 2:** Effect of test compounds on Calu-3 cells viability after incubation with 0.2% test solutions for 60 min.

S/N	Test Compounds	Chemical Class	European Union Class	Calu-3 %Viability ± SD
1	PEG400	Alcohol	NI	91.5 ± 4.6
2	3-Methoxy-1,2-propanediol	Alcohol	NI	92.6 ± 4.1
3	Glycerol	Alcohol	NI	85.6 ± 8.7
4	PEG600	Alcohol	NI	85.9 ± 13.4
5	2-Methyl-1-pentanol	Alcohol	NI	95.0 ± 13.1
6	Anhydrous ethanol	Alcohol	NI	99.1 ± 3.9
7	Cyclohexanol	Alcohol	R41	89.9 ± 14.3
8	Tween20	Surfactant	NI	57.1 ± 8.1
9	Sodium dodecyl sulphate USP	Surfactant	R36	4.3 ± 0.1
10	Triton X-100	Surfactant	R36	3.5 ± 0.5
11	Cetylpyridinium bromide	Surfactant	R41	8.1 ± 1.1
12	Toluene	Heterocyclic	NI	85.7 ± 13.0
13	Imidazole	Heterocyclic	R41	106.9 ± 10.8
14	Methyl isobutyl ketone	Ketone	NI	73.2 ± 7.4
15	Acetone	Ketone	R36	100.4 ± 13.7
16	Sodium hydroxide	Inorganic chemical	R41	4.6 ± 0.4
17	Sodium oxalate	Carboxylic acid salt	R41	65.4 ± 4.0
18	4-Fluoroaniline	Amine	R41	114.9 ± 5.9

European Union irritation classification: NI, non-irritant; R36, irritating to eye; R41, risk of serious damage to eyes.

The result obtained after the cells were incubated with 1.0% *w*/*v* of test solutions for 60 min (Table 3) showed that with the exception of 2-methyl-1-pentanol (2.9% ± 0.2%) all the alcohols including PEG400 (92.2% ± 15.9%); 3-methoxy-1,2-propanediol (90.7% ± 4.3%); glycerol (86.5% ± 7.0%); PEG600 (81.7% ± 10.8%); anhydrous ethanol (90.3% ± 8.0%); cyclohexene (78.7% ± 11.7%) maintained viabilities greater than 50%. All the surfactants, Tween 20 (43.9% ± 2.5%); sodium dodecyl sulphate USP (9.0% ± 0.1%); Triton X-100 (4.5% ± 0.1%); cetylpyridinium bromide (5.4% ± 0.6%) had viabilities less than 50%. Both heterocyclic compounds, toluene (109.8% ± 6.6%) and imidazole (95.4% ± 2.9%), as well as the ketones, methyl isobutyl ketone (90.3% ± 7.1%) and acetone (95.4% ± 16.5%) had viability values of more than 50%. For sodium hydroxide (14.9% ± 1.2%), 1.0% *w*/*v* of the compounds reduced the cell viability of the cells more than 0.2% solutions following 60 min exposure while for 4-fluoroaniline (75.6% ± 10.9%), 0.2% *w*/*v* of the compounds reduced the cell viability of the cells more than 1.0% solutions following 60 min exposure.

**Table 3:** Effect of test compounds on Calu-3 cells viability after incubation with 1.0% test solutions for 60 min.

S/N	Chemical	Chemical Class	European Union Class	Calu-3 %Viability ± SD
1	PEG400	Alcohol	NI	92.2 ± 15.9
2	3-Methoxy-1,2-propanediol	Alcohol	NI	90.7 ± 4.3
3	Glycerol	Alcohol	NI	86.5 ± 7.0
4	PEG600	Alcohol	NI	81.7 ± 10.8
5	2-Methyl-1-pentanol	Alcohol	NI	2.9 ± 0.2
6	Anhydrous ethanol	Alcohol	NI	90.3 ± 8.0
7	Cyclohexanol	Alcohol	R41	78.7 ± 11.7
8	Tween20	Surfactant	NI	43.9 ± 2.5
9	Sodium dodecyl sulphate USP	Surfactant	R36	9.0 ± 0.1
10	Triton X-100	Surfactant	R36	4.5 ± 0.1
11	Cetylpyridinium bromide	Surfactant	R41	5.4 ± 0.6
12	Toluene	Heterocyclic	NI	109.8 ± 6.6
13	Imidazole	Heterocyclic	R41	95.4 ± 2.9
14	Methyl isobutyl ketone	Ketone	NI	90.3 ± 7.1
15	Acetone	Ketone	R36	95.4 ± 16.5
16	Sodium hydroxide	Inorganic chemical	R41	14.9 ± 1.2
17	Sodium oxalate	Carboxylic acid salt	R41	64.5 ± 11.5
18	4-Fluoroaniline	Amine	R41	75.6 ± 10.9

European Union irritation classification: NI, non-irritants; R36, irritating to eye; R41, risk of serious damage to eyes.

Table 4 summarized the results of cells exposed to 0.2% and 1.0% *w*/*v* test solutions after 60 min exposure according to the ECVAM protocol. At 0.2% *w*/*v* solution, there were 5 false negatives (irritants falsely predicted as non-irritants). These compounds include cyclohexanol, imidazole, acetone, sodium oxalate, and 4-fluoroaniline. Four of these compounds were neither alcohol nor surfactant. There was no false positive (non-irritant falsely predicted as irritant) recorded. The major difference between the results of incubations of test solutions (0.2% and 1.0% *w*/*v*) for 60 min was two false positive results (Tween 20 and 2-Methyl-1-pentanol) obtained at 1.0% *w*/*v*. Table 4 shows that 0.2% test solutions gave a better prediction than 1.0%. Cells exposed to 1.0% solutions for 60 min resulted in five false negatives (cyclohexanol, imidazole, acetone, sodium oxalate, 4-fluoroaniline) (Table 4). Two false positives (Tween 20 and 2-Methyl-1-pentanol) were also observed.

**Table 4:** Comparison of validation data, based on test compound concentration and 60 min exposure to Calu-3 cells.

European Union Classification	Calu-3 Result			
	0.2% Test Solutions		1.0% Test Solutions	
	Irritants	Non-Irritants	Irritants	Non-Irritants
Non-Irritants (9)	0	9	2	7
Irritants (9)	4	5	4	5

The specificity, concordance and sensitivity for 0.2% *w*/*v* and 60 min incubation were 100%, 72% and 44%, respectively (Table 5). All non-irritants were correctly predicted and had cell viabilities beyond 85% except for Tween 20, which had a viability of 57.1% ± 8.1%. The irritants (R36, R41) that were correctly predicted had viabilities less than 9.0%, while irritants falsely predicted as non-irritants (false negatives) had percent viabilities beyond 65%. Six (PEG400, 3-Methoxy-1,2-propanediol, glycerol, PEG600, 2-Methyl-1-pentanol, anhydrous ethanol) out of the seven alcohols tested were correctly predicted. A similar observation was seen within the surfactant group where all four compounds (1 non-irritant and 3 irritants) were correctly predicted. The specificity, concordance and sensitivity for 1.0% *w*/*v* test compounds exposed to the Calu-3 cells for 60 min were 78%, 61% and 44% respectively (Table 5). Most (78%) of the non-irritants were correctly predicted. The two false positives have cell viabilities of 43.9 ± 2.5 and 2.9 ± 0.2 for Tween 20 and 2-Methyl-1-pentanol, respectively. The irritants (R36, R41) that were correctly predicted (sodium dodecyl sulphate USP, Triton X-100, cetylpyridinium bromide, sodium hydroxide) had viabilities less than 17% while irritants falsely predicted as non-irritants (false negatives) had viabilities of between 64% and 96%.

**Table 5:** Comparison of validation parameters (sensitivity, specificity, concordance) based on test compound concentration and 60 min exposure to Calu-3 cells. Comparison of results of Calu-3 cells treated with 0.2% and 1.0% test solutions for 60 min.

Groups	Sensitivity *	Specificity **	Concordance ***
1.0% at 60 min treatment	44%	78%	61%
0.2% at 60 min treatment	44%	100%	72%

* Sensitivity, the percentage of irritants correctly predicted. This was obtained by dividing the number of correctly predicted irritants by the total number of irritants; ** Specificity, the percentage of non-irritants correctly predicted. This was obtained by dividing the number of correctly predicted non-irritants by the total number of non-irritants; *** Concordance, the percentage of chemicals correctly predicted. This was obtained by dividing the number of correctly predicted compounds by the total number of compounds.

### 3.2. Discussion

The bovine corneal opacity and permeability (BCOP), Hen’s Egg Test on chorio-allantoic membrane (HET-CAM), chorioallantoic membrane vascular assay (CAMVA), isolated rabbit eye (IRE), isolated chicken eye (ICE), slug mucosal irritation (SMI) [12], and the reconstituted human corneal epithelial (HCE) methods [27,28] have been investigated as experimental models for mucosal irritation and toxicity screening. The BCOP assay method is one of the leading alternative assays to the Draize test and has been accepted by many regulatory agencies since 2009 [5]. The SMI was developed as a general test method for the nasal, buccal, and vaginal mucosal surfaces irritation and has been used for mucosal tolerance testing of pharmaceutical formulations and ingredients [10].

In this study we demonstrated that the Calu-3 cell culture model is a potentially useful cell line for investigating respiratory mucosa irritation. The sensitivity, specificity and concordance of our data compared favorably well to BCOP and SMI irritation models (Table 6). Our data showed that the range of values for each of the investigated assay parameter (sensitivity, specificity, concordance) were comparable to other methods: 75%–81% (BCOP), 68%–100% SMI (mucus endpoint), and 44%–100% (Calu-3 cell line; 0.2% at 60 min exposure), respectively. Furthermore, our model showed a concordance of 72%, which is slightly higher than that of the SMI model (68%), but lower than BCOP method (79%–81%).

**Table 6:** Comparison of sensitivity, specificity and concordance of different assay methods used in prediction of mucosal toxicity.

Methods	Sensitivity %	Specificity %	Concordance%	Source
BCOP	75–84	79–81	79–81	[29]
SMI (mucus endpoint)	75	100	68	[4]
Calu-3 cell model (0.2% at 60 min)	44	100	72	

Regarding the various test compounds, results from alcohols in our study were comparable to the data from the SMI model using mucus production as irritation endpoint. The non-irritating alcohols were correctly predicted as non-irritants whereas the irritating alcohols were under-predicted. Historically, the irritation potential of alcohols is difficult to predict. *In vivo*, irritating alcohols induced no increased mucus production in SMI model [12]. Alcohols/polyols have a tendency to introduce false results in the BCOP assay [29]. Results of validation studies conducted by the Interagency Coordinating Committee on the Validation of Alternative Methods (ICCVAM) for BCOP assay showed that false negative rates for alcohols and solids range from 42% to 100% depending on the hazard classification system [27]. Furthermore, alcohols and esters (including volatile substances, such as isopropanol, ethanol, methyl acetate, or ethyl acetate) are reported to have a relatively low predictive capacity compared to that of other substances [30].

In our studies, the four surfactants (Tween 20, sodium dodecyl sulfate, Triton^®^-X 100 and cetylpyridinium bromide) that were investigated were correctly predicted (100% concordance). In the SMI model, Adriaens and Remon (2002) reported 2 correct predictions (Tween 20, cetylpyridinium bromide) and 2 over-predictions (sodium dodecyl sulfate, Triton-X 100). The result shows that our model performed well on surfactants just like Hen’s Egg Test Chorioallantoic Membrane (HET-CAM) [29]. Similarly, methyl isobutyl ketone was wrongly classified in SMI but was correctly identified as non-irritant in our model. Furthermore, acetone gave a false negative both in SMI and in our model.

The same measure of specificity (100%), which was recorded both in SMI and Calu-3 cell models, means that both models are comparable when used to test non-irritants. The concordance of the BCOP test method with regard to each of the three classification systems (European Union (EU), EPA, and GHS) ranged from 79% to 81%. The false positive and false negative rates ranged from 19% to 21% and 16% to 25%, respectively [29]. Compared with BCOP, our model recorded a higher specificity with lower sensitivity (Table 6). A comparison of Draize eye test and BCOP reported 84.6% concordance with specificity and sensitivity being more than 84%. All the false negatives recorded were solids whereas most of the false positives were liquids, indicating that the physical state of the substance under investigation affects the result [31].

The higher specificity (100%) observed for our model implies that unlike the BCOP test (79–81) method, we were able to identify non-irritant compounds correctly, irrespective of its class. For a compound to be irritating to the cells, the compound must diffuse into the cells. We used Log *p* values comparison to assess which of the compounds had difficulty diffusing into the cells. The Log *p* values range for the test compounds was +4.15 to −1.76. For optimal permeation, an ideal compound generally has a Log *p* value of between 1 and 4 [31]. The non-irritating compounds have optimal Log *p* values; most of the non-irritating alcohols have negative values. All the non-irritating non-alcoholic compounds (cyclohexanol, sodium dodecyl sulphate USP, Triton X-100, cetylpyridinium bromide, toluene, methyl isobutyl ketone) with optimal Log *p* were correctly predicted except 4-fluoroaniline. Most of the non-alcoholic compounds with false negative results (imidazole, acetone, sodium oxalate) have negative Log *p* values. These suggest that the alcohols may not have penetrated the cell membranes in adequate quantities and it is possible that the false predictions associated with the alcohols may be due to their Log *p* values.

The wide disparity in the sensitivity values between BCOP (75%–84%) and Calu-3 cell model (44%) suggest that more work is required to improve the sensitivity of the Calu-3 model. Sensitivity disparity may be related to the fact that the test compounds affected cells viability via different mechanisms such as physical or interfacial mechanisms, hypertonicity, solvent-based solubilization, chelation, and membrane fluidization. Better sensitivity may be achieved by altering the Calu-3 cell culture method (e.g., using air-liquid interface or 3-D culture methods) or by using other toxicity indices other than MTT for estimating toxicity endpoint.

## 4. Conclusions

In conclusion, we validated the Calu-3 cell line as a tool for screening respiratory mucosal irritation and toxicity. It is our hope that further work will improve the sensitivity of this model and that, sometime in the future, this approach will be used for high throughput screening of respiratory mucosa irritation and toxicity. However, as the data reported in this study were solely based on MTT assay, additional studies are needed using other toxicity-/irritation-indicating methods to confirm the observed trend.
